# Predicting phenotype transition probabilities via conditional algorithmic probability approximations

**DOI:** 10.1098/rsif.2022.0694

**Published:** 2022-12-14

**Authors:** Kamaludin Dingle, Javor K. Novev, Sebastian E. Ahnert, Ard A. Louis

**Affiliations:** ^1^ Department of Chemical Engineering and Biotechnology, Cambridge University, Cambridge CB2 1TN, UK; ^2^ Department of Computing and Mathematical Sciences, California Institute of Technology, Pasadena, CA 91125, USA; ^3^ Department of Mathematics and Natural Sciences, Centre for Applied Mathematics and Bioinformatics (CAMB), Gulf University for Science and Technology, 32093, Kuwait; ^4^ Department of Physics, Rudolf Peierls Centre for Theoretical Physics, Oxford University, Oxford OX1 2JD, UK

**Keywords:** genotype–phenotype maps, evolution, complexity, algorithmic probability

## Abstract

Unravelling the structure of genotype–phenotype (GP) maps is an important problem in biology. Recently, arguments inspired by algorithmic information theory (AIT) and Kolmogorov complexity have been invoked to uncover *simplicity bias* in GP maps, an exponentially decaying upper bound in phenotype probability with the increasing phenotype descriptional complexity. This means that phenotypes with many genotypes assigned via the GP map must be simple, while complex phenotypes must have few genotypes assigned. Here, we use similar arguments to bound the probability *P*(*x* → *y*) that phenotype *x*, upon random genetic mutation, transitions to phenotype *y*. The bound is $P(x\to y)≲2^{-a\tilde{K}(y|x)-b}$, where $\tilde{K}(y|x)$ is the estimated conditional complexity of *y* given *x*, quantifying how much extra information is required to make *y* given access to *x*. This upper bound is related to the conditional form of algorithmic probability from AIT. We demonstrate the practical applicability of our derived bound by predicting phenotype transition probabilities (and other related quantities) in simulations of RNA and protein secondary structures. Our work contributes to a general mathematical understanding of GP maps and may facilitate the prediction of transition probabilities directly from examining phenotype themselves, without utilizing detailed knowledge of the GP map.

## 1. Introduction

An important challenge within theoretical biology is understanding the structure of *genotype–phenotype (GP) maps*, which dictate how gene sequences are translated into different biological forms, functions and traits, known as phenotypes. Elucidating GP map structure is essential to a proper understanding of evolution [1], because, while random mutations occur at the genetic level, the effects of mutations occur at the level of the phenotype and therefore depend on the GP map structure.

Several common properties of GP maps have been identified [2,3], such as a strong bias in terms of how many genotypes are assigned to each phenotype [4–7] and high degrees of robustness to genetic mutations [8–11]. Significantly, the GP map structure has been shown to strongly influence the trajectories and outcomes of evolution: computer simulations of the evolution of RNA secondary structures (SS) [12,13], protein complexes [6,14], genetic circuits [15], among others [16] have shown that even in the presence of natural selection the bias arising from the GP map structure can influence and even dominate outcomes. More significantly, for naturally occurring RNA shapes [9,12,17–22] and protein quaternary structures [23], the frequency in nature of different molecular shapes can be predicted from GP map biases. The way genotypes are associated with phenotypes via the GP map is also known to limit and constrain evolutionary accessibility of phenotypes [24–26].

Despite the importance of GP maps and the observed common properties across a variety of example maps, the theoretical underpinnings for these observations are not well developed. A recent approach to mathematically studying GP map structure is to use arguments inspired by *algorithmic information theory* (AIT) [27–29], a field of computer science that studies the information content and complexity of discrete patterns, structures and shapes. Based on these arguments in [23,30,31], it was shown that the estimated information content, or *Kolmogorov complexity*, of a phenotype shape is closely connected to the probability that the shape appears on random sampling of genotypes. Moreover, high-probability phenotype shapes were shown to be simple, and more complex shapes were exponentially less probable, leading to the discovery of *simplicity bias* (SB) in GP maps. Interestingly, this complexity approach has enabled predicting the frequency with which biomolecule shapes appear in databases of natural biomolecules [23,32]. More broadly, many studies have shown that employing AIT as a theoretical framework combined with estimates of Kolmogorov complexity can be fruitful in natural sciences. Example applications include in thermodynamics [33–35], understanding the regularity of the laws of physics [36], entropy estimation [37,38], classifying biological structures [39], evolution theory [40,41], networks [42,43], in addition to data analysis [44–46] and time series analysis [47,48], among others [49].

Here, we extend the earlier work on SB in GP maps by utilizing information complexity arguments to predict phenotype transition probabilities: we use AIT as a theoretical framework to derive probability–complexity relations to make predictions about the probability *P*(*x* → *y*) that phenotype *y* appears upon the introduction of a single random genetic point mutation in a genotype coding for the phenotype *x*. We show that *P*(*x* → *y*) is fundamentally related to the conditional complexity of *y* given *x*, which measures how much information is required to produce phenotype *y* given access to *x*. We derive an upper bound equation and test it computationally on models of RNA and protein secondary structure, finding good quantitative accuracy.

## 2. Null models for transition probabilities

### 2.1. Problem set-up

We will focus on GP maps that have some large (finite) number *N*_*g*_ of discrete genotype sequences, a large number *N*_*p*_ of possible phenotypes, each of which is designable (i.e. has at least one genotype). Further, we will assume that there are many more genotypes than phenotypes, such that 1 ≪ *N*_*p*_ ≪ *N*_*g*_, which is a common property of well-studied GP maps [3]. The GP map will be denoted *f*. The phenotypes are assumed to be some kind of discrete pattern, sequence or shape, or at least one that could be discretized. For example, a protein quaternary structure can be represented as a discrete graph of nodes [32], and a continuous chemical concentration–time curve can also be discretized in a number of ways [30]. We assume the map is deterministic, such that each genotype maps consistently to only one phenotype.

As an example of such a GP map, we can take the RNA sequence-to-secondary structure map for which a genotype of length *L* featuring four nucleotides (*A*, *U*, *C* and *G*) has *N*_*g*_ = 4^*L*^ possible sequences. Also, *N*_*p*_ ∼ 1.8^*L*^ [20] so that 1 ≪ *N*_*p*_ ≪ *N*_*g*_ even for modest *L*, and the phenotypes can be represented as discrete sequences, because RNA SS are commonly given in a dot-bracket form, consisting of a string of *L* symbols defining the bonding pattern of the molecule. In contrast to this RNA example, we are not considering GP maps with only few phenotypes, such as whether a patient does or does not have cancer, for which there are only two possible phenotypes, and ‘has cancer’ is not a discrete pattern or shape.

We will write *P*(*x*) for the probability that phenotype *x* appears when uniformly randomly sampling a genotype out of the full collection of *N*_*g*_ genotypes. *P*(*x*) will be called the *global frequency* of *x*. Although the average probability will be 1/*N*_*p*_, possibly for some phenotypes *P*(*x*) ≫ 1/*N*_*p*_ due to bias, and also possibly *P*(*x*) ≪ 1/*N*_*p*_ for some phenotypes. The *neutral set* (NS) of *x* is the set of genotypes that map onto *x*. If we pick one random genotype *g* from the NS of *x* and make a random single-point mutation such that we have a new genotype *g*′, we will call the resulting phenotype *y*. It is possible that phenotypes *x* and *y* are the same or different because *g*′ may possibly be in the NS of *x*.

If *x* = *y*, then we designate the mutation as neutral. We will define *P*(*x* → *y*) as the transition probability that a randomly selected genotype from the NS of *x*, upon a single random mutation, yields the phenotype *y*. Note that we will still use the word ‘transition’ even if *x* = *y*. A phenotype is called *robust* to mutations if the probability that *y* = *x* is high, i.e. the phenotype typically remains unchanged after a random mutation. The high robustness of phenotypes to genetic mutations is essential to life, and evolution (at least as we know it) would not be able to proceed without it [8,50]. The origin of high robustness has been seen as something of a mystery, however [50]. In addition, it has been noticed in several GP maps that robustness scales with the logarithm of the global frequency of a phenotype, as opposed to scaling with the global frequency itself, which would be expected from a random null model. The cause of this general logarithmic scaling is presently not fully explained, although an abstract model of GP maps has been used to study this property [51]. In a future study, we intend to look in detail at robustness.

The problem of estimating *P*(*x* → *y*) is the main focus of this study, and in particular relating this quantity to the relative information contents of *x* and *y*.

### 2.2. Null models of transition probability

How can we estimate the transition probability *P*(*x* → *y*)? If we have access to data recording the frequency of transitions in simulations, then we could directly estimate *P*(*x* → *y*) from those data by counting the number of times *x* transitioned to *y* as a fraction of all transitions starting with *x*. It may also be possible to arrive at an estimate of *P*(*x* → *y*) by examining details of the map and the particular phenotype *x*. However, what we are interested in here is a general method for predicting *P*(*x* → *y*), that does not rely on using past frequency data, or details of the map. Indeed, we are interested in general properties of GP maps, which will both help to develop a theory of GP maps and also be useful for other maps for which we neither have data nor a clear understanding of exactly how the phenotype arises from the genotypes. In a sense, we are interested in *a priori* prediction for *P*(*x* → *y*), which is based only on the patterns in *x* and *y*. At first sight, this goal may not appear possible because *P*(*x* → *y*) *will* depend on the details of the map. However, we will argue in this work that even without knowing details of the map and without recourse to historical frequency data, non-trivial predictions for the transition probabilities can be derived. In another sense, we are interested in establishing a good null model for *P*(*x* → *y*), which could serve as a starting point for predictions about transitions. In this connection, we now consider some possible null models and weigh up their merits.

Perhaps the simplest null model expectation is that the transition probability is

2.1 $$P(x\to y)=\frac{1}{N_{p}},$$

which corresponds to a maximum entropy estimate, assigning a uniform probability to each possible outcome *y*. However, this has a limitation, which is that a common property of GP maps is bias (described earlier), and so it seems reasonable to expect some degree of non-uniformity in *P*(*x* → *y*). Further, the high levels of robustness discussed earlier do not accord with this uniform distribution model. From here onwards, we will assume that the distribution *P*(*x* → *y*) over the possible values of *y* is strongly non-uniform (biased).

Another simple null model for *P*(*x* → *y*) was proposed in [13],

2.2 P(x→y)≈P(y)

for *y* ≠ *x*. This null model prediction is correct if genotypes are randomly assigned to phenotypes, with no correlations between genotypes or phenotypes. While the approximation in equation (2.2) is straightforward and was observed to be quite accurate on average [13], it also has limitations. Firstly, as pointed out in [30], many GP maps have fixed and somewhat simple rule-sets by which genotypes are assigned to phenotypes (technically, they are *O*(1) complexity maps). Hence, these maps do not randomly assign genotypes, but assign them with a definite structure and pattern, which is likely to produce some clear patterns in genotype architecture. Secondly, it is intuitively reasonable that phenotype *x* will transition to some *y*, which is similar or even the same as *x*. The logic being that one single-point mutation represents a small change to the genotype, and consequently, a small change to the phenotype appears to be a rational null assumption. Of course this assumption that GP maps are roughly ‘continuous’ in the mathematical sense of the word does not always hold, because some (well-chosen) mutations may drastically change the phenotype, but nonetheless the assumption has intuitive appeal and may typically hold. Greenbury *et al.* [50] have also suggested that transitions are more likely to be similar phenotypes (with the caveat that it must be possible for enough genotypes to be sampled), arguing via genetic correlations in GP maps. Hence, equation (2.2) has limitations as a null model.

To improve on equation (2.2), we would like to incorporate the ‘similar phenotypes’ notion in a formal way, which will lead to a new null model that we propose. To do this, we first need to survey some pertinent theoretical background.

## 3. Algorithmic information theory

### 3.1. Kolmogorov complexity

Developed within theoretical computer science, *algorithmic information theory* [27–29] (AIT) connects computation, computability theory and information theory. The central quantity of AIT is *Kolmogorov complexity*, *K*(*x*), which measures the complexity of an individual object *x* as the amount of information required to describe or generate *x*. More formally, the Kolmogorov complexity *K*_*U*_(*x*) of a string *x* with respect to a universal Turing machine (UTM) [52] *U*, is defined [27–29] as follows:

3.1 $$K_{U}(x)=\min_{\;p}\{|p|\;:\;U(p)=x\},$$

where *p* is a binary program for a prefix (optimal) UTM *U* and |*p*| indicates the length of the (halting) program *p* in bits. Due to the invariance theorem [49] for any two optimal UTMs *U* and *V*, *K*_*U*_(*x*) = *K*_*V*_(*x*) + *O*(1) so that the complexity of *x* is independent of the machine, up to additive constants. Hence, we conventionally drop the subscript *U* in *K*_*U*_(*x*) and speak of ‘the’ Kolmogorov complexity *K*(*x*). Despite being a fairly natural quantity, *K*(*x*) is uncomputable, meaning that there cannot exist a general algorithm that for any arbitrary string returns the value of *K*(*x*). Informally, *K*(*x*) can be defined as the length of a shortest program that produces *x*, or simply as the size in bits of the compressed version of *x*. If *x* contains repeating patterns like *x* = 1010101010101010, then it is easy to compress, and hence, *K*(*x*) will be small. On the other hand, a randomly generated bit string of length *n* is highly unlikely to contain any significant patterns and hence can only be described via specifying each bit separately without any compression, so that *K*(*x*) ≈ *n* bits. *K*(*x*) is also known as *descriptional complexity*, *algorithmic complexity* and *program-size complexity*, each of which highlights the idea that *K*(*x*) measures the amount of information required to describe or generate *x* precisely and unambiguously.

An important quantity for our present investigation is the *conditional complexity*, *K*(*y*|*x*), defined as follows:

3.2 $$K(y|x)=\min_{\;p}\{|p|\;:\;U(x,p)=y\},$$

i.e. the minimum length of a program *p* such that a UTM *U* generates string *y*, given *x* and *p* as an input. Less formally, *K*(*y*|*x*) quantifies how many bits of information are required to generate *y*, given that we have access to *x*.

More details and technicalities can be found in standard AIT references [49,53–55] and a book aimed at natural scientists [56].

### 3.2. Algorithmic probability

In AIT, Levin’s [57] coding theorem establishes a fundamental connection between *K*(*x*) and probability predictions. Building on Solomonoff’s discovery of *algorithmic probability* [27,58], Levin’s coding theorem [57] states that

3.3 $$P(x)=2^{-K(x)+O(1)},$$

where *P*(*x*) is the probability that (prefix optimal) UTM *U* generates output string *x* on being fed random bits as a program. Thus, high-complexity outputs have exponentially low probability, and simple outputs must have high probability. *P*(*x*) is also known as the *algorithmic probability* of *x*.

The *conditional coding theorem* [59] states that the probability *P*(*y*|*x*) of generating string *y* with UTM *U* given access to string *x* as side information is expressed as follows:

3.4 $$P(y|x)=2^{-K(y|x)+O(1)}.$$

Notice that outputs with high probability here must have low conditional complexity *K*(*y*|*x*). To have *K*(*y*|*x*) low means that either *y* is simple itself, or it is similar to *x*. To see this, consider that if *y* is simple, then *K*(*y*) is low, then so too is *K*(*y*|*x*), hence *P*(*y*|*x*) is high. Also, if *y* is similar to *x*—e.g. they share common subsequences—then *K*(*y*|*x*) will be low, and *P*(*y*|*x*) will be high.

### 3.3. Simplicity bias

Equation (3.3) as well as many other AIT results cannot be straightforwardly applied to typical natural systems of interest in engineering and sciences due to the fact that (i) Kolmogorov complexity is uncomputable and so cannot be calculated exactly; (ii) the theory is asymptotic, valid only up to *O*(1) terms; (iii) the theory is largely based on UTMs, which are seldom present in nature; and (iv) the coding theorem assumes infinite purely uniform random programs, which do not exist in nature.

Despite these points, several lines of reasoning motivate using AIT to make predictions while being aware of the limitations of this practice. We call this kind of theoretical work ‘AIT-inspired’ arguments. See electronic supplementary information III (A and B) for more discussion on this.

Adopting the methodology of AIT-inspired arguments, Dingle *et al.* [30] studied coding theorem-like behaviour and algorithmic probability for (computable) real-world input–output maps. This led to their observation of SB, governed by the following equation:

3.5 $$P(x)\leq 2^{-a\tilde{K}(x)-b},$$

where *P*(*x*) is the (computable) probability of observing output *x* on random choice of inputs, and $\tilde{K}(x)$ is the approximate Kolmogorov complexity of the output *x*. In words, SB means complex outputs from input–output maps have lower probabilities, and high probability outputs are simpler. The constants *a* > 0 and *b* can be fit with little sampling and often even predicted without recourse to sampling [30].

Examples of systems exhibiting SB are wide ranging and include molecular shapes such as protein structures and RNA [23], outputs from finite-state machines [31], as well as models of financial market time series and ordinary differential equation (ODE) systems [30], among others. A full understanding of exactly which systems will, and will not, show SB is still lacking, but the phenomenon is expected to appear in a wide class of input–output maps, under fairly general conditions. See electronic supplementary material III (C) for more on this.

## 4. Simplicity bias in transitions

### 4.1. Simplicity bias: conditional form

Just as for the original coding theorem, the conditional coding theorem in equation (3.4) cannot be directly applied to practical real-world systems, such as making estimates for phenotype transition probabilities. So we derive (electronic supplementary material III (D and E)) a conditional form of the SB equation equation (3.5), which we subsequently apply to phenotype transition probabilities: The conditional form is

4.1 $$P(x\to y)\leq 2^{-K(y|x,f)+O(1)}.$$

### 4.2. Complexity of the genotype–phenotype map

The complexity of the GP map *f* is an important quantity. If the map *f* is allowed to have a high-complexity value, then *f* could be chosen such that *P*(*x* → *y*) takes arbitrary values, and hence, it will be very hard to predict transition probabilities. Fortunately, many GP maps are not random, but in fact have simple (low-complexity) fixed rule-sets for determining how genotypes are assigned to phenotypes [30]. See electronic supplementary material III (F) for more details.

If we restrict our attention to GP maps for which *f* is of fixed complexity, i.e. *K*(*f*) = *O*(1), then this means that *K*(*y*|*x*, *f*) ≈ *K*(*y*|*x*) so that equation (4.1) becomes

4.2 $$P(x\to y)\leq 2^{-K(y|x)+O(1)},$$

and we see that this upper bound depends only on the phenotypes *x* and *y*. So the complexity of the map *f* is an important quantity which either does or does not allow predictions to be made just using conditional complexities.

### 4.3. Approximation of the upper bound

Because Kolmogorov complexity is uncomputable, in practice, we use approximations, such as real-world compression algorithms [49] (see also electronic supplementary material III (B) for more on this). Following the approximation and scaling arguments of [30], we can write an approximate form of equation (4.2),

4.3 $$P(x\to y)≲2^{-a\tilde{K}(y|x)-b},$$

which is a weaker form of the full AIT conditional coding theorem [59] given in equation (3.4). The term $\tilde{K}(y|x)$ is an approximation of the conditional Kolmogorov complexity *K*(*y*|*x*), which we will calculate according to the Lempel–Ziv [60] complexity estimate used earlier [30,31] and also scale the complexity values so that $0≲\tilde{K}(y|x)≲\log_{2}(N_{p})$ as described in the methods in electronic supplementary material I (A). To estimate the conditional complexity $\tilde{K}(y|x)$, we employ the approximation (as used earlier [61]) that

4.4 $$\tilde{K}(y|x)\approx \tilde{K}(xy)-\tilde{K}(x),$$

where $\tilde{K}(xy)$ is the compressed length of the concatenation of strings *x* and *y*. For example, if x=ABC and y=XY, then xy=ABCXY. Note that for true prefix Kolmogorov complexity, the relation *K*(*y*|*x*) ≈ *K*(*x*, *y*) − *K*(*x*) only holds to within logarithmic terms [49], but that is close enough for our purposes. Note that the terms *K*(*x*, *y*) and *K*(*xy*) are quantitatively very close, especially if the lengths of *x* and *y* are the same. Hence, we make the approximation that they are equal.

The constant *a* may depend on the map, but not on the phenotype *x*. If the complexity $\tilde{K}(y|x)$ is scaled properly (electronic supplementary material I (A)), then *a* = 1 is the default prediction. Otherwise, *a* might have to be fit to the data. The main requirement for scaling properly is having a reasonably accurate estimate of the number *N*_*y*_(*x*) of phenotypes *y* such that *P*(*x* → *y*) > 0, i.e. the number of accessible phenotypes via a single-point mutation from *x*. If *N*_*y*_(*x*) is known *a priori* or can be estimated *a priori*, then *N*_*y*_(*x*) can be used for *a priori* prediction of *P*(*x* → *y*). Otherwise, if random genotype sampling is employed, then simply counting the number of different *y* phenotypes observed in sampling is one way to estimate *N*_*y*_(*x*). Naturally, this counting method will be more accurate for larger samples and may produce very low underestimates of *N*_*y*_(*x*) for small sample sizes.

The constant *b* has default value *b* = 0 [30], but can also be found by fitting to the data if necessary. Looking at the examples of SB presented in the literature to date, it appears that *b* = 0 often works very well.

It follows that in practice, provided some reasonable estimate of the number of accessible phenotypes *N*_*y*_(*x*) is known and hence complexities are scaled well, then equation (4.3) reduces further to the practically applicable relation

4.5 $$P(x\to y)≲2^{-\tilde{K}(y|x)}$$

allowing transition probabilities to be made just based on phenotype conditional complexities.

### 4.4. The bound is close with high probability

On the basis of arguments in [31], we expect the upper bound equation (4.3) (and also equation (4.5)) to be tight for *x*, *y* pairs, which are generated by random genotypes. That is, for a phenotype *x* generated by a random genotype, and *y* subsequently arising from a random mutation, we expect $P(x\to y)\approx 2^{-a\tilde{K}(y|x)-b}$ with high probability, as opposed to the right-hand side being only a loose upper bound.

On the other hand, the ubiquity of low-complexity, low-probability outputs [31,62] suggests that for many *y* we may have $P(x\to y)\ll 2^{-a\tilde{K}(y|x)-b}$. Such phenotypes *y* are those that have low conditional complexity, yet at the same time appear with low probability due to map-specific constraints and biases. See [62] for an in-depth discussion of this low-complexity, low-probability phenomenon.

### 4.5. Size of the genotype alphabet and number of mutations

For the bound of equation (4.3) to have stronger predictive value on point mutations, we suggest that the size of the genotype alphabet *α* should be small. This is not a very onerous condition and is in fact quite naturally satisfied. Further, the number of mutations should be approximately 1. See electronic supplementary material III (G) for more on these conditions.

### 4.6. When is *P*(*y*) a good predictor of *P*(*x* → *y*)?

From AIT, we know that almost all pairs of phenotypes *x* and *y* share almost no information, in other words, *K*(*x*, *y*) ≈ *K*(*x*) + *K*(*y*), so that

4.6 K(y|x)≈K(y).

From this, we can infer that for almost all pairs of phenotypes *x* and *y*, the conditional complexity *K*(*y*|*x*) in equation (4.1) can be replaced with just *K*(*y*), and so the equation becomes

4.7 $$P(x\to y)≲2^{-K(y)}$$

for almost all *y*.

The preceding argument suggests that for most outputs *y*, the phenotype *x* may be largely irrelevant in estimating the probabilities *P*(*x* → *y*). However, this statement comes with the caveat that nearly all the probability mass is likely to be associated with only a small fraction of the possible outputs, and those for which *K*(*y*|*x*) is low. See electronic supplementary material III (H) for more discussion and details.

### 4.7. Predicting which of *P*(*x* → *y*_*i*_) or *P*(*x* → *y*_*j*_) is higher

Another quantity that may be predicted using the preceding theory is the ratio of probabilities for transitioning from one phenotype to different alternative phenotypes. In this section, we describe a method for such predictions, which we test numerically below.

Call *y*_*i*_ the resulting phenotype after a single-point mutation to a randomly chosen genotype in the NS of *x*. Call *y*_*j*_ the resulting phenotype after a single-point mutation to another independently chosen random genotype, also in the NS of *x*. We can use the preceding theory to predict which of the two phenotypes *y*_*i*_ and *y*_*j*_ has a higher probability directly from complexity estimates. This is interesting because it is often valuable to know whether *P*(*x* → *y*_*i*_) > *P*(*x* → *y*_*j*_) or *P*(*x* → *y*_*i*_) < *P*(*x* → *y*_*j*_), rather than trying to guess the exact values of *P*(*x* → *y*_*i*_) and *P*(*x* → *y*_*j*_). Fortunately, constants *a* and *b* are not required for predicting this via equation (4.3) because only the relative values of $\tilde{K}(y_{i}|x)$ and $\tilde{K}(y_{j}|x)$ determine whether $2^{-a\tilde{K}(y_{i}|x)-b}$ or $2^{-a\tilde{K}(y_{j}|x)-b}$ is larger. So even if we could not estimate *a* or *b* accurately, we could still make a prediction about which phenotype is more likely to arise through a point mutation of *x*. See electronic supplementary material I (B) for more details.

### 4.8. Predicting *P*(*x* → *y*_*i*_)/*P*(*x* → *y*_*j*_) ratios

Beyond predicting which has higher probability, we can also try to predict the value of the ratio of probabilities of transitioning from one phenotype to different alternative phenotypes. The ratio is also related to how confident we are in predicting which of the probabilities *P*(*x* → *y*_*i*_) and *P*(*x* → *y*_*j*_) is higher: a higher ratio means more confidence in the prediction.

Because both *y*_*i*_ and *y*_*j*_ are randomly generated, we expect both *P*(*x* → *y*_*i*_) and *P*(*x* → *y*_*j*_) to be close to the bound of equation (4.3), with high probability [30,31]. Therefore we can use an approximate equality assumption to predict the ratio as follows:

4.8 $$\begin{array}{cc} \frac{P(x\to y_{i})}{P(x\to y_{j})} & \approx \frac{2^{-a\tilde{K}(y_{i}|x)-b}}{2^{-a\tilde{K}(y_{j}|x)-b}}=2^{-a\tilde{K}(y_{i}|x)}/2^{-a\tilde{K}(y_{j}|x)}\\ \Rightarrow \log_{10}\frac{P(x\to y_{i})}{P(x\to y_{j})} & \approx a\log_{10}(2)(\tilde{K}(y_{j}|x)-\tilde{K}(y_{i}|x)) \end{array}\},$$

where *b* is irrelevant, so that even if *b* is not known, the prediction is unaffected. Recalling from earlier that we set *a* = 1 yields $\log_{10}(2)(\tilde{K}(y_{j}|x)-\tilde{K}(y_{i}|x))$ as the predictor for the log_10_ ratio of the probabilities. If the scaling is not done correctly, then *a* ≠ 1, and therefore, the predictor will not be as accurate, but instead off by a constant factor. In this case, we still have a relative measure of how confident we are about the prediction, where larger values of $\log_{10}(2)(\tilde{K}(y_{j}|x)-\tilde{K}(y_{i}|x))$ are associated with higher-confidence predictions.

### 4.9. Distribution of conditional complexities

Equation (4.3) states that higher-probability phenotypes must have low conditional complexity, so in this sense, we have derived a ‘conditional simplicity bias’. A related but different question is, upon choosing a random genotype in the NS of *x*, and introducing a random mutation, what conditional complexity value is most likely? More generally, what kind of distribution should we expect for *K*(*y*|*x*)? This is not a trivial question, because on the one hand, the upper bound on transition probabilities decays exponentially with increasing *K*(*y*|*x*), which would suggest that lower complexities are more likely. On the other hand, from AIT, we expect the number of patterns with higher complexity to grow exponentially with increasing complexity and hence increase with *K*(*y*|*x*), which would suggest that higher complexities are more likely. However, how the actual number of conditional complexities grows for a specific GP map system may not reflect the AIT expectation exactly.

In [23] (see also [48]), it was argued that to a first approximation, these two exponential trends should cancel each other out, leading to a ‘flat’ complexity distribution. On the basis of these arguments, we suggest that perhaps a flat distribution will also be seen for *K*(*y*|*x*). Predicting the distribution of complexities is somewhat difficult due to the fact that for a ‘flat’ distribution the two exponential trends must precisely cancel out, while exponential trends can easily magnify small errors in approximation. The distribution of complexities should be investigated in the future work.

## 5. Empirical phenotype transition probabilities

### 5.1. Main predictions

Before analysing some example GP maps, we recap the main predictions and equations discussed in this work:

(a) High transition probabilities *P*(*x* → *y*) will be associated with phenotypes which either are similar to *x* or are very simple.
(b) Transition probabilities will conform to the upper bound $P(x\to y)≲2^{-a\tilde{K}(y|x)-b}$ as shown in equation (4.3), with *a* = 1 and *b* = 0 as default expectations.
(c) Some phenotypes will have probabilities close to the upper bound, while many may be low-complexity, low-probability outputs far below the bound.
(d) For most phenotypes *y*, *K*(*y*|*x*) ≈ *K*(*y*) and especially if *K*(*x*) is low, but *K*(*y*|*x*) ≪ *K*(*y*) for some *y*.
(e) We can predict which of the two phenotypes is more likely to arise just by comparing their conditional complexities, betting on the simpler one having higher probability.
(f) We can predict the actual ratio of these two probabilities.

In the following sections, we computationally test the applicability of these predictions to RNA and protein SS.

### 5.2. Computational experiment: RNA secondary structure

RNAs are important and versatile biomolecules that act as information carriers, catalysts and structural components, among other roles. Similarly to DNA, RNA is composed of a sequence of nucleotides, which can contain four possible nucleobases, *A*, *U*, *C* and *G*. RNA molecules typically fold up into well-defined SS, which denote bonding patterns in the molecule. The SS shape is determined by the underlying sequence, but at the same time, the sequence-to-SS map is highly redundant with many different sequences adopting the same SS. RNA SS has been very well studied in biophysics and computational biology because it is a biologically relevant system, but at the same time, fast and accurate computational algorithms exist for predicting SS from sequences. Here, we use the popular Vienna RNA [63] package for predicting SS, which utilizes a thermodynamics-based algorithm.

To test our predictions, we randomly generated an RNA sequence of length *L* = 40 nucleotides and computationally predicted its SS .(((...)))..((((.((..((....)).))))))...., which we will denote by *x*. We chose *L* = 40 because computational efficiency requires a comparatively short sequence, but on the other hand, complexity–probability connections are more pronounced for longer RNA [30]. To estimate *P*(*x* → *y*), we need to generate a large sample of sequences within the neutral space of *x*, that is, different sequences that each have *x* as their SS. To generate the sequences, we used the site-scanning method of [64]. Afterwards, we introduced a single random point mutation for each neutral sequence and recorded the resulting SS. More details are given in the methods described in electronic supplementary material I (C).

**Figure 1.**
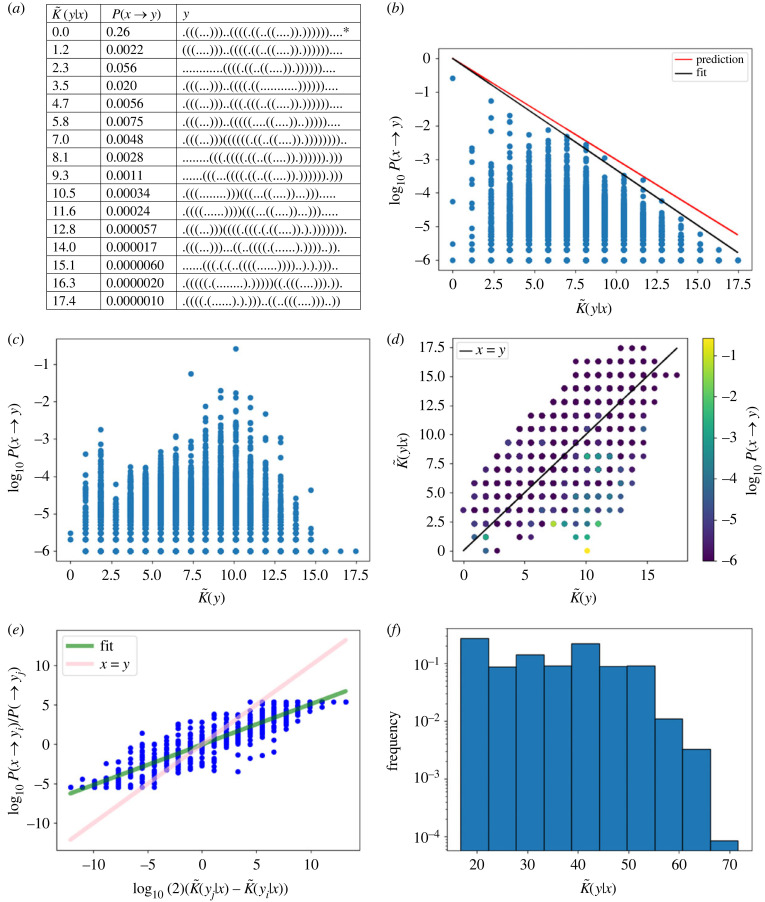
RNA secondary structure transition probabilities for a sequence of length *L* = 40 nucleotides. The starting phenotype is x=.(((...)))..((((.((..((....)).)))))).... and transitions result from choosing random genotypes in the NS of *x*, and introducing a single random mutation to each genotype. (*a*) Table illustrating the highest-probability SS for each conditional complexity value. The starting phenotype *x* is marked with an asterisk (*), and *P*(*x* → *y*) is just the robustness since *x* = *y*. (*b*) Transition probabilities *P*(*x* → *y*) decrease exponentially with increasing conditional complexity $\tilde{K}(y|x)$, upper bound of equation (4.3) depicted in black. The highest-probability SS is the same phenotype as *x*. The predicted upper bound (red) and fitted bound (black) are close. (*c*) The unconditional complexity $\tilde{K}(y)$ does not predict the transition probabilities well. (*d*) $\tilde{K}(y|x)\approx \tilde{K}(y)$ for most *y*, leading to a positive linear correlation between values, compared with equation (4.6). (*e*) Ratios of probabilities correlate strongly with differences in conditional complexity, partially according with equation (4.8). (*f*) The histogram of conditional complexity values shows a roughly flat distribution (on a log scale), but with some slight bias towards simplicity.

Figure 1*a* shows the highest-probability SS for each conditional complexity value found in the dataset generated in this way, as well as the estimated probability of transitioning from the starting phenotype *x* to all of these alternative phenotypes. In figure 1*b*, we see that, as expected, there is strong bias in transition probabilities with a decay in the upper bound to *P*(*x* → *y*). The black line is a fit to the data (*a* = 1.1 and *b* = 0) added to highlight the upper bound. Figure 1*b* also shows the predicted upper bound (red line) based on *a* = 1 and *b* = 0; this prediction is impressive, given that it is based on just the output complexities themselves. Note that there are several low-probability structures for which our measure $\tilde{K}(x|y)$ gives zero, even though they are slightly different to the reference structure. This is most likely caused by two effects: firstly, by the lack of very fine resolution in our approximate complexity measure, and secondly, by our neglecting the *O*(1) terms. Figure 1*c* presents the same data as figure 1*b*, except that the horizontal axis is $\tilde{K}(y)$ instead of $\tilde{K}(y|x)$, and it is apparent that $\tilde{K}(y)$ does not provide a good predictor of the probabilities. This demonstrates that the conditional complexities are needed, not just the complexity $\tilde{K}(y)$. Figure 1*d* is a scatter plot of $\tilde{K}(y)$ vs $\tilde{K}(y|x)$ and, as expected, there is a linear (but noisy) correlation between these two quantities (Pearson *r* = 0.64, *p*-value < 10^−6^). It is also interesting to see that, as expected, a small number of phenotypes have conditional complexities $\tilde{K}(y|x)$ much lower than the unconditional complexity $\tilde{K}(y)$ and those tend to be the higher-probability outputs (as can be seen from the colouring of the datapoints by log probability).

Turning to the prediction of which of two phenotypes has higher probability, we find that with probability-weighted sampling, the accuracy is a striking 86%, and with uniform sampling, the rate is still impressive at 79%. (Recall that probability-weighted sampling refers to when genotypes are uniformly randomly sampled, and hence phenotypes appear with frequencies according to their probabilities. Uniform sampling refers to when each phenotype is sampled with equal probability.) Extending this, figure 1*e* depicts not just predictions of which is higher, but of the ratios themselves. Although the fit does not match the *x* = *y* prediction line, there is nonetheless a strong correlation (Pearson *r* = 0.91, *p*-value < 10^−6^) between the ratios of the probabilities and the differences in complexities. This means that although the slope is not very well predicted, the expected close connection between the complexity values and the probabilities holds. Recall that the inaccuracy in the slope predictions for figure 1*b*,*e* results primarily from a lack of precision in estimating the value of *a*, which depends on knowing the number of possible phenotypes *y* such that *P*(*x* → *y*) > 0. The fact that the actual slope is flatter than the predicted one is presumably due to the following: the value of $\log_{10}(2)(\tilde{K}(y_{j}|x)-\tilde{K}(y_{i}|x))$ will be large when *y*_*j*_ is (conditionally) complex and *y*_*i*_ is simple. Therefore, *y*_*j*_ is unlikely to be far from the upper bound, while very simple phenotypes can be very far from the upper bound, compared with the low-complexity, low-probability phenomenon [31,62]. Hence, the value of *P*(*x* → *y*_*i*_)/*P*(*x* → *y*_*j*_) is likely to be an underestimate, rather than an overestimate, which will tend to make the slope flatter.

Finally, figure 1*f* presents a histogram of conditional complexities, showing that their distribution is roughly flat on a log scale as tentatively predicted, but it also exhibits some mild bias towards lower-complexity phenotypes.

In electronic supplementary material II, we numerically study the impact of the complexity of the starting phenotype *x*, showing that the mean value in the difference between $\tilde{K}(y|x)$ and $\tilde{K}(y)$ is small for simple *x* as expected and grows for more complex *x*. Further, in electronic supplementary material IV, we provide an additional example RNA plot, depicting results similar to those shown in figure 1.

### 5.3. Computational experiment: protein secondary structure

Proteins are biomolecules that form the fundamental building blocks of organisms. A protein is composed of one or more macromolecular chains, that in living organisms, it typically contains 20 types of amino acid residues. Similar, to RNA, a protein will fold into a particular spatial structure, which is determined by the specific amino acid sequence. There is redundancy in that many different sequences can have the same fold [65]. The overall three-dimensional arrangement of a protein’s polypeptide chain in space is known as its tertiary structure, while protein SS refers to the local conformation of the polypeptide backbone. SS is a key to protein folding [66], and the genotype-to-phenotype map between primary and secondary structure has not received much attention in the literature to date. At the level of detail, we are concerned with determining that a protein’s SS is equivalent to specifying whether each amino acid residue in the chain is involved in a coil (*C*), sheet (*E*) or helix (*H*) structure. Hence, the SS of a protein of length *L* is also a sequence of length *L*, but with only three possible letters (*C*, *E*, *H*) at each site.

Predicting the full three-dimensional structure of a protein was until recently an open problem, but it is now feasible via machine learning algorithms such as AlphaFold [67]. However, it remains very computationally taxing and potentially unreliable for sequences not related to the natural sequences used to train the underlying machine learning algorithm. In contrast, the machine learning-based Porter 5 algorithm provides accurate and relatively fast predictions of protein SS [68]. Predicting the structure of mutants remains challenging both for algorithms such as AlphaFold [69] and for SS ones like Porter 5. Here, we make the implicit assumption that Porter 5 captures the large-scale properties of the mapping between protein primary and secondary structure sufficiently accurately that our computational study grants insight into the system. Such insight is particularly valuable considering that it is impractical to survey a similar number of mutants experimentally.

Like with the RNA example, choosing the length *L* of the protein under study is a balance between computational cost and accuracy: if longer proteins are used, then the number of possible SS grows exponentially, making it hard to get accurate estimates of probabilities, as the latter require the same SS to appear multiple times. On the other hand, very short proteins are less biologically relevant, and the complexity measures and theory are expected to work worse for them than for longer sequences. Here, we balance these considerations by studying a complementarity-determining region (CDR) with *L* = 20, specifically CDRH3 from the heavy chain of a human monoclonal SARS-CoV antibody (Protein Data Bank (PDB) [70] ID: 6WS6 [71]). Antibody complementarity-determining (hypervariable) regions are critical in the recognition of antigens and extreme sequence variation in them allows antibodies to bind to a nearly limitless range of antigens. The particular antibody under the study potently neutralizes the SARS-CoV-2 virus [72]. The CDRH3 region is especially important for antigen recognition [73,74], and its conformation is not restricted to a small set of canonical structures, unlike those of the other CDRs [73–75]. Note also that because Porter 5 is a machine learning-based algorithm, it is only expected to be accurate for sequences similar to natural sequences on which it was trained. Hence, the need to use a naturally occurring protein, rather than a completely random sequence, for which Porter 5 may yield inaccurate SS predictions. The SS predicted by Porter 5 for the chosen CDRH3 is x=EEECCCCCCCCCCCCCCCCC, and this SS was highly accurate (85%) when compared with experimentally derived SS, EEECCCCCCCCCCCCCCEEE. We used site scanning [64] to generate a large number of different sequences within the neutral space of *x*. We then generated all mutant sequences reachable via a point mutation in DNA for a subset of the sample of neutral genotypes and used Porter 5 to predict their SS (more details in electronic supplementary material I (D)).

**Figure 2.**
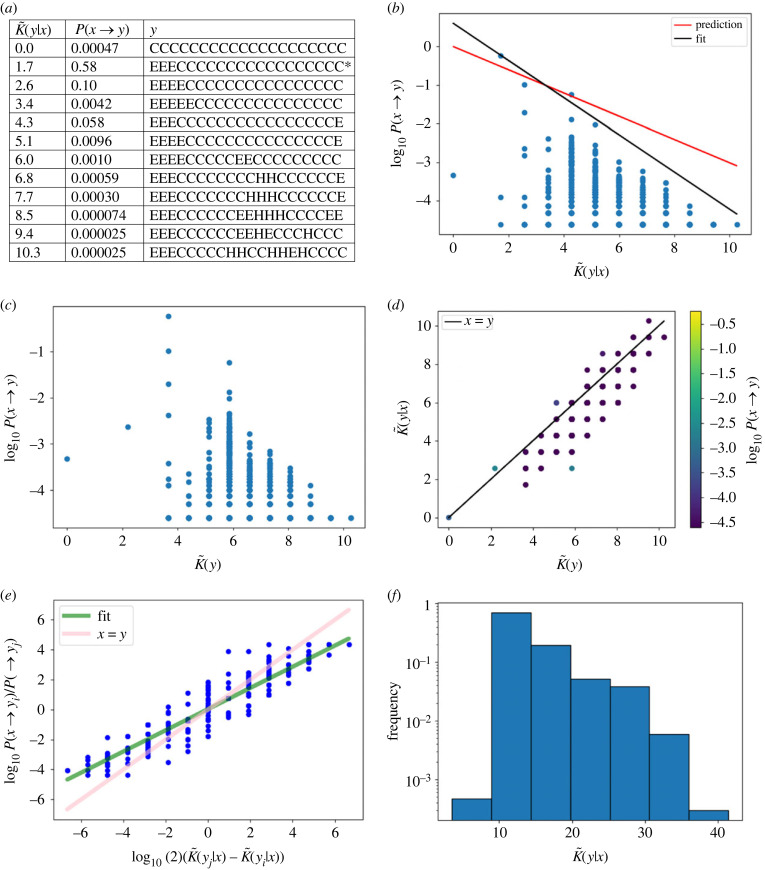
Protein secondary structure transition probabilities for CDRH3 from the heavy chain of a human monoclonal SARS-CoV antibody (PDB ID: 6WS6 [71]), length *L* = 20, *x* =EEECCCCCCCCCCCCCCCCC. (*a*) Table illustrating the highest-probability SS for each conditional complexity value. The starting phenotype *x* is marked with an asterisk (*), and *P*(*x* → *y*) is just the robustness since *x* = *y*. (*b*) Transition probabilities *P*(*x* → *y*) decrease exponentially with increasing conditional complexity $\tilde{K}(y|x)$. The phenotype with the lowest conditional complexity is CCCCCCCCCCCCCCCCCCCC. The phenotype with the highest probability is *y* = *x*. The predicted upper bound (red) is close to the fitted bound (black, compared with equation (4.3)). (*c*) The unconditional complexity $\tilde{K}(y)$ predicts the transition probabilities nearly as well as $\tilde{K}(y|x)$, presumably because *x* has low complexity (see electronic supplementary material II). (*d*) $\tilde{K}(y|x)\approx \tilde{K}(y)$ for most *y*, leading to a positive linear correlation between values. (*e*) Ratios of probabilities correlate strongly with differences in conditional complexity. (*f*) The histogram of conditional complexity values shows a bias towards simpler proteins with lower-complexity values.

Figure 2*a* shows the most frequent SS for each conditional complexity value found in the dataset generated via site scanning, as well as the estimated probability of transitioning from the starting phenotype *x* to all of these alternative ones. In figure 2*b,* we see a strong bias in transition probabilities with a linear decay in the upper bound to *P*(*x* → *y*), as predicted. The black line is a fit to the data (*a* = 1.6 and *b* = −2), added to highlight the upper bound. Figure 2*b* also shows the predicted upper bound based on *a* = 1 and *b* = 0 (red line); this prediction is useful, but not as accurate as for RNA *L* = 40. Figure 2*c* presents the same data as shown in figure 2*a*, except that $\tilde{K}(y)$ is on the horizontal axis instead of $\tilde{K}(y|x)$. Interestingly, $\tilde{K}(y)$ is quite similar to $\tilde{K}(y|x)$, which is probably due to the fact that the original starting phenotype *x* is very simple (electronic supplementary material II). The scatter plot of $\tilde{K}(y)$ vs $\tilde{K}(y|x)$ in figure 2*d* shows that, as expected, there is a linear correlation between these two quantities (Pearson *r* = 0.87, *p*-value < 10^−6^). It is interesting that the correlation is stronger than for the RNA example, and this again can be rationalized by the fact that *x* is very simple. There do not appear to be any phenotypes for which $\tilde{K}(y|x)$ is much smaller than $\tilde{K}(y)$. With probability-weighted sampling, the accuracy of predicting which of two phenotypes has higher probability is very high at 79%, and with uniform sampling is 77%. Figure 2*e* shows predictions for the ratio of the probabilities for transitioning to different phenotypes versus the difference between their estimated complexity in log scale. The latter displays a strong correlation (Pearson *r* = 0.93, *p*-value < 10^−6^). Finally, figure 2*f* presents a histogram of conditional complexities, showing a stronger bias towards lower-complexity phenotypes than the data for RNA. In electronic supplementary material IV, an additional electronic supplementary figure shows accurate predictions for another protein SS example.

## 6. Discussion

We have studied the problem of predicting the probability of transition between phenotypes *x* and *y*, *P*(*x* → *y*), from the perspective of algorithmic information theory (AIT), and specifically algorithmic probability estimates. We derived an upper bound on *P*(*x* → *y*), which depends on the conditional complexity of phenotype *y* given *x*. The derivations were motivated by the observation that the assignment of genotypes to phenotypes is highly structured, and the expectation that the constraints of information theory should therefore have predictive value in genotype-to-phenotype maps. Upon testing our various predictions on RNA and protein secondary structure examples, we found good quantitative agreement between the theory and the simulations.

The benefit of developing this theoretical approach is that it allows predictions to be made about transition probabilities for GP maps in which only phenotypes are observed and little or no knowledge about the map is available. This approach is also relevant to uncovering general common properties of GP maps, which are important for the advancement of the currently underdeveloped field of theoretical biology [76].

In this study, we restricted our attention to one aspect of the structure of the GP, without including evolutionary dynamic effects resulting from mutation rates, strength of selection, population size or others that would be relevant in biology. Therefore, we leave these to the future work. It is interesting, however, that studies of natural RNA shapes [21,22] and the shapes of protein complexes [23] have shown that GP map biases alone can be very good predictors of natural biological shape frequencies (see also [41,77–79] for more on different types of biases and evolutionary outcomes). Therefore, it may be that the transition probability biases discussed here, resulting from conditional complexity constraints, are strong enough that their stamp is still observable even in natural data. We suggest that an interesting follow-on study to ours would be to test this with natural bioinformatic databases.

In this study, we have tested our transition probability predictions on two biologically important GP maps, namely, RNA and protein sequence-to-structure maps. However, in these GP maps, the connection between genotypes and phenotypes is quite direct and fairly simple. Furthermore, for computational reasons, we studied only short RNA and proteins. In biology, many GP maps have a less direct connection between genotypes and phenotypes, and it remains a possibility that our probability predictions do not work well for such complicated maps. We leave the exploration of the limits of applicability of our theory for future work. Having said this, it is noteworthy that other researchers have empirically observed a tendency for genetic mutations to favour simpler morphologies, specifically in teeth [80], embryo [81] and leaf shapes [82]. We believe that our results help rationalize these observations within a general theoretical framework. In addition, these empirical observations made in the context of complicated and realistic biological maps may suggest that our theory can be applied in more complex GP maps.

Another area of potential applicability of our transition probability predictions is in genetic algorithms for optimization. Indeed, Hu *et al.* [83], as well as others [84,85], have studied optimization problems and shown that some target phenotypes are harder to find than others, not only because of having a low global frequency but also due to local mutational connections. In the future work, it would be interesting to assess if these mutational connections are related to conditional complexity, as our theory would suggest.

Robustness to genetic mutations is an important property for organisms [8], but a general explanation for the high levels of robustness observed in GP maps has been lacking [50]. Our information theory perspective here relates to this question because we have seen that transitions to phenotypes which are similar to the starting phenotype tend to have high probability, and of course, a phenotype is most similar to itself. We intend to explore this in detail in a forthcoming study.

Other authors have used information theory for non-UTMs to derive some results which are related to the ones we derived here. For example, Calude *et al.* [86] developed bounds for finite-state machines (the simplest computing devices), and moreover, Merhav and Cohen [87] have derived similar probability bounds to ours directly in terms of Lempel–Ziv complexity. It would be interesting to see if these calculations for non-UTMs could be extended to GP maps. In electronic supplementary material III (A), we discuss in more detail the use of AIT arguments in science.

While the upper bound from equation (4.3) appears to work well in the simulations presented here, a main weakness in our predictions is that many phenotypes that have low conditional complexities $\tilde{K}(y|x)$ also have low probabilities. Because these phenotypes fall far below the upper bound, their precise probabilities are not well predicted by the theory. These low-complexity, low-probability patterns have been described as having low absolute information content, but due to map-specific biases, they are ‘hard’ for the map to make and hence have low probability [31]. The origins and nature of these types of patterns have been recently studied [62], but a full understanding of them and knowledge regarding how to improve probability predictions of these have not yet been achieved. Despite the challenge of low-complexity, low-probability patterns, we were still able to make high-accuracy upper bounds on phenotype probabilities as well as high-accuracy (approx. 80%) predictions about which of two phenotypes is more likely, just using complexity values.

The quantitatively accurate predictions we describe here motivate further investigation of the use of AIT-inspired predictions in biology, evolution and other natural sciences.

## Data Availability

The data for the proteins analysis is available from the public repository Protein Data Bank (PDB) with ID: 6WS6. For the RNA analysis, we did not use natural data, rather we generated random sequences. Code is available from the electronic supplementary material [88].
